# Depletion of Cholesteryl Esters Causes Meibomian Gland Dysfunction-Like Symptoms in a *Soat1*-Null Mouse Model

**DOI:** 10.3390/ijms22041583

**Published:** 2021-02-04

**Authors:** Igor A. Butovich, Amber Wilkerson, Seher Yuksel

**Affiliations:** 1 Department of Ophthalmology, University of Texas Southwestern Medical Center, Dallas, TX 75390-9057, USA; amber.wilkerson@utsouthwestern.edu (A.W.); seher.yuksel1@gazi.edu.tr (S.Y.); 2 The Graduate School of Biomedical Sciences, University of Texas Southwestern Medical Center, Dallas, TX 75390-9057, USA

**Keywords:** cholesteryl esters, chromatography, lipidomics, mass spectrometry, Meibomian gland, Meibomian gland dysfunction, meibogenesis, *Soat1*/SOAT1

## Abstract

Previous studies on ablation of several key genes of meibogenesis related to fatty acid elongation, omega oxidation, and esterification into wax esters have demonstrated that inactivation of any of them led to predicted changes in the meibum lipid profiles and caused severe abnormalities in the ocular surface and Meibomian gland (MG) physiology and morphology. In this study, we evaluated the effects of *Soat1* ablation that were expected to cause depletion of the second largest class of Meibomian lipids (ML)—cholesteryl esters (CE)—in a mouse model. ML of the *Soat1-*null mice were examined using liquid chromatography high-resolution mass spectrometry and compared with those of *Soat1^+/−^* and wild-type mice. Complete suppression of CE biosynthesis and simultaneous accumulation of free cholesterol (Chl) were observed in *Soat1-*null mice, while *Soat1^+/−^* mutants had normal Chl and CE profiles. The total arrest of the CE biosynthesis in response to *Soat1* ablation transformed Chl into the dominant lipid in meibum accounting for at least 30% of all ML. The *Soat1-*null mice had clear manifestations of dry eye and MG dysfunction. Enrichment of meibum with Chl and depletion of CE caused plugging of MG orifices, increased meibum rigidity and melting temperature, and led to a massive accumulation of lipid deposits around the eyes of *Soat1*-null mice. These findings illustrate the role of *Soat1*/SOAT1 in the lipid homeostasis and pathophysiology of MG.

## 1. Introduction

Exocrine Meibomian glands (MG) [1] are lipid-producing structures that biosynthesize and excrete meibum via a holocrine mechanism [2]. Meibum has been shown to play a central role in protecting the ocular surface from the environment by forming, together with lacrimal gland secretions, a protective structure called tear film [3,4,5]. Many similarities between biochemistry and physiology of human MG and those of several animal species have been reported [6,7]. Meibomian lipids (ML) that are the major part of meibum are very diverse. Two major lipid classes—very, extremely, and ultra-long chain wax esters (WE) and cholesteryl esters (CE)—represent, accordingly, about 40% and 30% of all meibum [8,9]. Other lipid classes that make up for the rest of the secretion include (*O*)-acylated ω-hydroxy fatty acids (OAHFA [10], up to 5% or so), cholesteryl esters of OAHFA (Chl-OAHFA), and diacylated α,ω -diols (DiAD) [6]. The exact quantitation of the latter two classes has not been performed yet because of the lack of proper chemical standards. The minor components of meibum include free cholesterol (Chl < 1%), triacylglycerols (TAG < 1%), free fatty acids (<1%), and remnants of ruptured cell membranes—phospholipids and sphingomyelins which are normally present at <0.1%. This mixture of lipids is quite complex as each family of lipids is represented by dozens of individual lipid species, which all seem to be critical for meibum to effectively fulfill its protective role.

In 2016–2017, a concept of *meibogenesis* was introduced [7,11] to provide a unified view on the role of specific genes and enzymes in the biosynthesis of meibum. Key genes of meibogenesis were identified and linked to provide a template for future studies of lipid metabolism in MG [12]. Recently, in complete accordance with those predictions, such functional links have been established in direct experiments. Indeed, interruption of the FA elongation cycle in MG caused by inactivating mutations in *Elovl1* [13] and *Elovl3* [14] genes, or inhibition of FA ω-oxidation by inactivation of *Cyp4F39* [15], or inactivation of *Awat2* [16,17], led to equally massive changes in meibum lipid profiles and MG and ocular surface physiology and morphology in mutant mice. Interestingly, many of those effects resembled human pathologies such as dry eye, MG dysfunction (MGD), blepharitis, and others. Thus, a direct link between FA elongation, oxidation, and esterification into WE and MG lipid homeostasis and various ocular pathologies has been established. 

However, the roles of the second largest group of ML—CE—have not been described. The goal of this study was to evaluate the effects of inactivating the mutation of a gene sterol *O*-acyltransferase-1 (*Soat1*) [18,19,20]—the most highly expressed gene of the Chl esterification pathway in MG of humans and mice [7,11,12]—on the CE profiles and ocular features of *Soat1^−/−^* and *Soat1^+/−^* mice.

## 2. Results

### 2.1. General Characterization of Soat1-Null Mice and Their Ocular Phenotype

**Figure 1:**
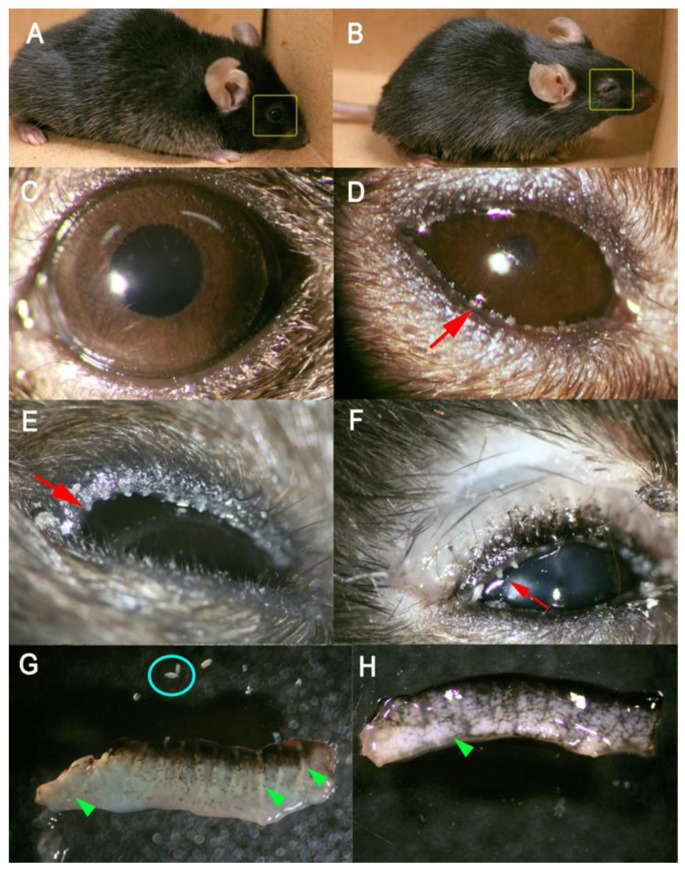
Characterization of ocular phenotype of *Soat1*-null mice. (**A**) Wild-type male mouse with normal fur and normal eye opening (yellow box). (**B**) *Soat1*-null male mouse with normal fur and slit eye opening (yellow box). (**C**) Slit lamp image of a normal mouse eye. (**D**) *Soat1*-null slit lamp image with meibum accumulations around the eye (red arrow). (**E**) Pouting of Meibomian gland orifices of a *Soat1*-null mouse (red arrow); (**F**) Thick meibum extruding from the orifices (red arrow) upon application of gentle pressure. (**G**) Excised *Soat1*-null tarsal plate with visible ducts and indistinct acini (green triangle) and solidified meibum accumulations (blue circle). (**H**) Wild-type tarsal plate with distinct acini (green triangle).

Pups and young adult *Soat1^−/−^* and *Soat1^+/−^* mice had no visible abnormalities in their skin and fur coating. However, *Soat1*-null pups had noticeably smaller eye fissures at 2 weeks of age, compared with their wild-type (WT) littermates. It is not known at this time whether this effect was congenital or a voluntary action/reflex action to the ocular surface irritation. The most noticeable ocular feature of the adult mutant mice was the slit eye phenotype (Figure 1A–D). The ellipticity of the eye openings [14] was calculated using the following Formula (1):

(1) $$e\;=\;\surd (1-b^{2}/a^{2})$$

where ***a*** and ***b*** are semi-major and semi-minor axes of the ellipse. For a perfectly round eye ***e*** would be 0, while for an oblong one, more than 0 but less than 1. The measured ellipticity ***e*** of *Soat1-*null mice was in the 0.92 ± 0.01 range (i.e., highly elliptical or elongated) while for the WT, between 0.57 ± 0.03 (rounder). Another way of geometrical characterization of the eye opening is to calculate the eye’s short and long axes ratio, which for a round eye would be 1, while for elongated one, between 0 and 1. For *Soat1-*null mice the ratio was found to be about 0.38, while for WT mice, around 0.85. Under microscope evaluation, tarsal plates (TP) of mutant mice had indistinct, almost atrophic or underdeveloped MG acini with severe pouting of most of the orifices. Other characteristic features of the phenotype included a thick, toothpaste-like consistency of *Soat1-*null meibum at physiological temperatures and accumulation of solidified lipid-like debris around the eye openings. The central ducts were plugged with amassed solidified meibum that protruded beyond the orifices (Figure 1E,F). At room temperature, the *Soat1*-null meibum was much more solid than the normal one, to the extent of being brittle. Applying even a light pressure on the eyelids or excised TP caused the meibum to be expelled from the central ducts as solid rice grain-like formations (Figure 1G). For comparison purposes, a healthy TP of a WT mouse is shown alongside (Figure 1H). Complete characterization of the ocular surface physiology of *Soat1-*null mice will be published elsewhere.

### 2.2. Inactivation of Soat1 Arrested Biosynthesis of Cholesteryl Esters in MG of Soat1-Null Mice

**Figure 2:**
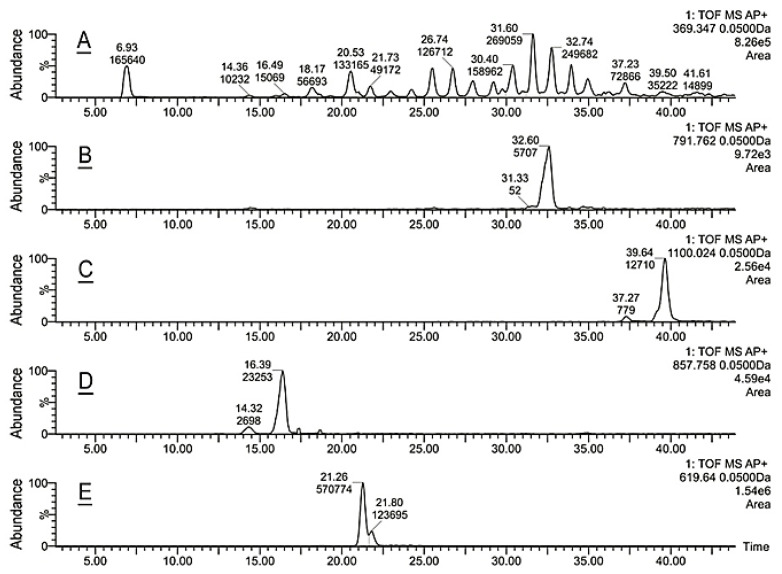
Lipidomic reverse phase chromatography/high-resolution mass spectrometry in atmospheric pressure chemical ionization mode (RP UPLC/APCI MS) analysis of normal mouse meibum in positive ion mode. (**A**) Extracted ion chromatogram of free Chl (a peak with a retention time 6.93 min) and CE (peaks with retention times between ~14 min and ~40 min) detected as a common analytical ion C_24_H_45_^+^. (**B**) Extracted ion chromatogram of C_28:1_-CE detected as (M + H)^+^ adduct C_55_H_99_O_2_^+^. (**C**) Extracted ion chromatogram of a Chl-OAHFA detected as (M + H)^+^ adduct C_75_H_135_O_4_^+^. (**D**) Extracted ion chromatogram of a proton adduct of a TAG detected as (M + H)^+^ adduct C_55_H_101_O_6_^+^. (**E**) Extracted ion chromatogram of a wax ester detected as (M + H)^+^ adduct C_42_H_83_O_2_^+^. Integrated peak areas (ion counts) are shown in the graphs below the retention times (in minutes). Normalized chromatograms are shown.

The lipid samples were analyzed using ultra-high performance reverse phase chromatography/high-resolution mass spectrometry in atmospheric pressure chemical ionization mode (RP UPLC/APCI MS) exactly as described in our recent publication [14,21]. A typical atmospheric pressure chemical ionization (APCI) experiment was conducted in the positive ion mode recording high-resolution mass spectra in the 100 to 2000 amu range. Extracted ion chromatograms (EIC) of analytes of interest were generated using a 50 mDa window, and the corresponding elution peaks were integrated using MassLynx’s “Integrate” routine to provide retention times and relative abundances of analytes in the samples. Representative EIC with retention times and peak areas for Chl and all CE (using signal *m/z* 369.35, elemental composition C_24_H_45_), a cholesteryl ester with C_28:1_ FA (using a signal of its proton adduct *m/z* 791.76; C_55_H_99_O_2_), a proton adduct of a Chl-OAHFA (*m/z* 1100.02; C_75_H_135_O_4_), a H^+^ adduct of triacylglycerol *m/z* 857.76 (C_55_H_101_O_6_), and a H^+^ adduct of a major wax ester C_42_H_83_O_2_ with *m/z* of 619.64 are shown in Figure 2A–E.

**Figure 3:**
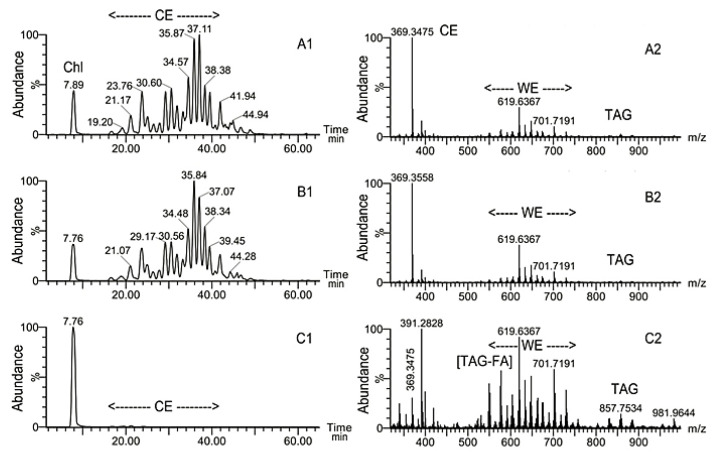
Effects of *Soat1* inactivation on Meibomian lipid profiles of mice. Panels **A1**,**B1**,**C1**: Extracted ion chromatograms of free Chl and CE detected as an ion with *m/z* 369.35; Panels **A2**,**B2**,**C2**: observation mass spectra of the samples. **A1**,**A2**—wild-type mouse; **B1**,**B2**—heterozygous mouse; **C1**,**C2**—homozygous mouse. Peak *m/z* 391.2828—unknown.

Inactivating mutation of *Soat1* led to an almost complete arrest of CE and Chl-OAHFA biosynthesis in *Soat1*-null mouse MG (Figure 3A1–C2). WT mice had a profile of CE that demonstrated a familiar (from previous publications [7,14,21]) distribution of CE species and a normal observation mass spectrum (Figure 3A1,A2). Surprisingly, *Soat1^+/−^* specimens produced no major changes in Chl, CE, and WE profiles (Figure 3B1,B2). However, a striking effect of the mutation was observed for *Soat1^−/−^* mice (Figure 3C1,C2): An almost complete loss of normal Meibomian-type CE occurred, with a sole peak of free Chl dominating the chromatogram. An observation mass spectrum of the sample (Figure 3C2), obtained by averaging spectra collected from 10 to 50 min into the chromatogram, confirmed a dramatic decrease in the analytical signal of CE, and revealed another anomaly—a multi-fold increase in the apparent abundances of TAG. However, all normal Meibomian WE (such as waxes with *m/z* 619.6367, 701.7191, and others) were still detectable. 

**Figure 4:**
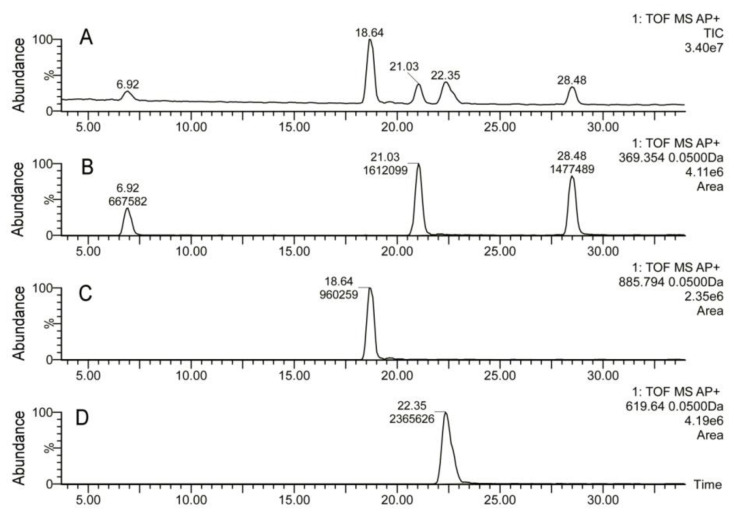
RP UPLC/APCI MS analysis of a series of equimolar lipid standards. (**A**) Total ion chromatogram of a 50-µM (each compound) mixture of free cholesterol (retention time 6.92 min), cholesteryl oleate (21.03 min), cholesteryl nervonate (28.48 min), triolein (18.64 min), and lignoceryl oleate (22.35 min). Panels (**B**–**D**)—extracted ion chromatograms of the standards, whose *m/z* values are indicated in the right corners of the panels, while the peak retention times and areas—near the top of each peak.

**Figure 5:**
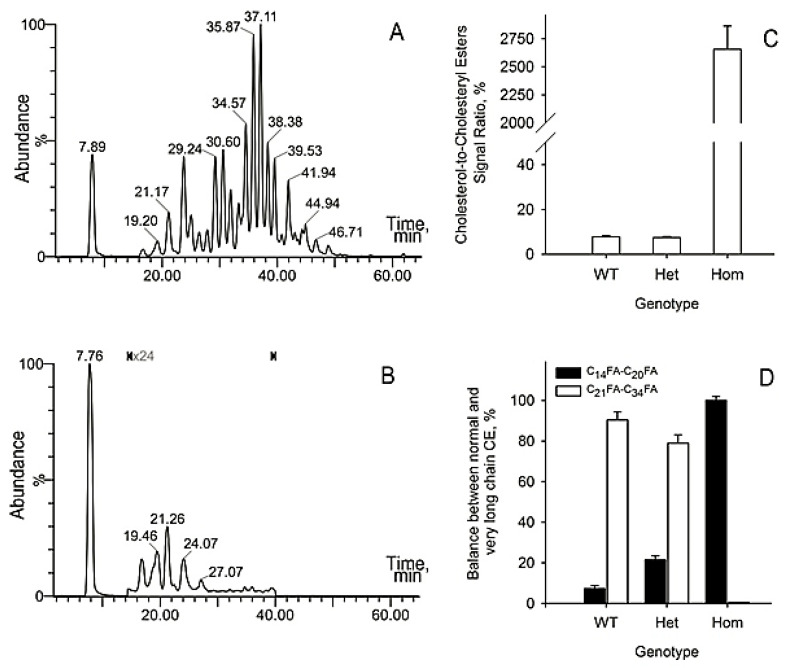
Inactivation of *Soat1* causes suppression of the biosynthesis of long chain cholesteryl esters and accumulation of free cholesterol and short chain cholesteryl esters in meibum. Cholesterol and its esters in wild-type (**A**) and *Soat1*-null (**B**) meibum. Note that the portion of the chromatogram between 14 and 40 min is magnified ×24 to make peaks visible on the graph (see black markings in the graph). Homozygous samples (*n* = 5) were statistically different from heterozygous ones (*n* = 7; *p* < 0.001). There were no statistically significant difference between heterozygous (*n* = 7) and wild-type samples (*n* = 5; *p* > 0.3). Effects of genotype on the cholesterol-to-cholesteryl esters ratios in meibum (**C**). Effects of *Soat1* ablation on the elongation patterns of Meibomian cholesteryl esters (**D**). All samples were statistically different from each other (*p* < 0.05). Note that formation of Chl-OAHFA was also severely inhibited. Results are shown as mean ± standard deviation values.

To quantitate the changes in lipid profiles, a series of lipid standards was prepared and studied as shown in Figure 4. An equimolar mixture of several representative lipids that were identified in mouse meibum—Chl, cholesteryl oleate (CO), cholesteryl nervonate (CN), triolein, and lignoceryl oleate (LO)—was prepared. The mixture was analyzed using RP UPLC/APCI MS in the same conditions as meibum samples. The signals of the analytes were plotted as EIC and integrated to determine their peak areas. It was found that the relative intensities of signals of Chl, CO, and CN, detected using their common fragment *m/z* 369.35, were 1.00, 2.21, and 2.41, correspondingly. This common ion was used to estimate the molar ratio of Chl and various CE in the samples. Based on our observations, the changes in Chl to CE ratios in response to the mutation were quantitated (Figure 5). In WT *Soat1^+/+^* mice, the Chl/CE ratio was within (7 ± 1)% for all tested animals. The ratio was virtually unchanged in *Soat1^+/−^* mice, but rose to >99% in *Soat1*-null mice. Simultaneously, the molecular profile of remaining <1% of CE was changed as well: All CE with FA chains longer than C_20_ were obliterated, while the shorter ones—C_16_ to C_20_—were still visible, albeit at very low CE/Chl ratios.

**Figure 6:**
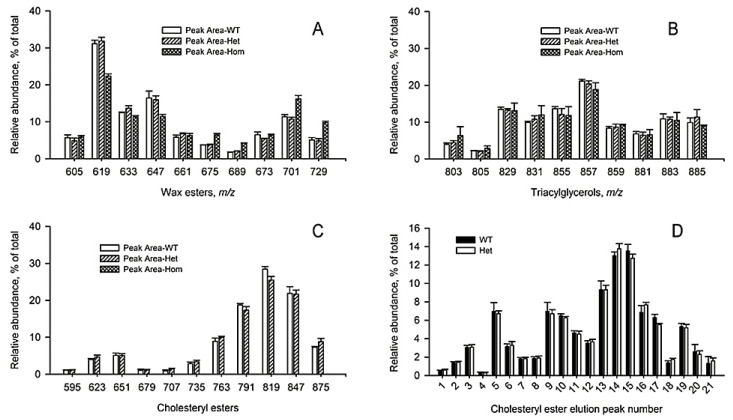
Targeted lipidomic analysis of wild-type (*n* = 5), *Soat1^+/−^* (*n* = 7), and *Soat1^−/−^* (*n* = 5) mouse meibum. Normalized results are shown as mean ± standard deviation values. For each genotype in each of the panels, total sum of lipids equals 100%. (**A**) Wax esters, (**B**) triacylglycerols, and (**C**) cholesteryl esters analyzed as (M + H)^+^ adducts; (**D**) cholesteryl esters analyzed as analytical ions *m/z* 369.35. The following assignments were made based on the *m/z* values and fragmentation patterns: C_10:0_ (1); C_12:0_- and C_14:1_-CE (2); C_14:0_ and C_16:1_ (3); C_15:0_ (4); C_16:0_- and C_18:1_-CE (5); C_17:0_-CE (6); C_18:0_- and C_20:1_-CE (7); C_19:0_-CE (8); C_20:0_- and C_22:1_-CE (9); C_21:0_-CE (10); C_22:0_- and C_24:1_-CE (11); C_23:0_-CE (12); C_24:0_- and C_26:1_-CE (13); C_25:0_-CE (14); C_26:0_- and C_28:1_-CE (15); C_27:0_-CE (16); C_28:0_- and C_30:1_-CE (17); C_29:0_ (18); C_30:0_- and C_32:1_-CE (19); C_34:1_-CE (20); unidentified (21). A sample chromatogram with corresponding peak assignments is shown in Supplemental Figure S1. Structures of relevant mouse Meibomian gland lipids were established and reported in our earlier publications [7,12,14].

Next, the changes in the balance of different lipid classes in response to the mutation were measured. When WE were visualized using their EIC and the peaks were integrated, no changes in the overall profiles of major WE in WT and *Soat1^+/−^* mice were detected (Figure 6A). However, complete ablation of *Soat1* caused a statistically significant decrease in monounsaturated WE with *m/z* 619.6389, 633.6545, and 647.6702, and an increase in longer chain monounsaturated WE *m/z* 675.7015 and diunsaturated WE *m/z* 701.7171 and 729.7484, which implies a possible connection between CE and WE pathways. The TAG profiles of all variants of mutant mice remained unchanged (Figure 6B).

Similar graphs for individual CE are shown in Figure 6C,D. Extracted ion chromatograms of ten major monounsaturated CE (MUCE; detected as proton adducts) were virtually identical for WT and *Soat1^+/−^* mice. The CE peaks of *Soat1-*null mice (of the C_10_ to C_18_ variety; illustrated in Figure 5B) were all below the low limit of quantitation and are not shown. When the common analytical ion *m/z* 369.35 was used to visualize all CE and Chl-OAHFA in the samples (Supplemental Figure S1), no differences in their elution profiles for WT and *Soat1^+/−^* mice were observed either. These observations made it possible to conclude that the CE and Chl-OAHFA molecular profiles of *Soat1^+/−^* mice did not change in response to partial *Soat1* ablation, but complete ablation of *Soat1* terminated the CE and Chl-OAHFA biosynthesis.

**Figure 7:**
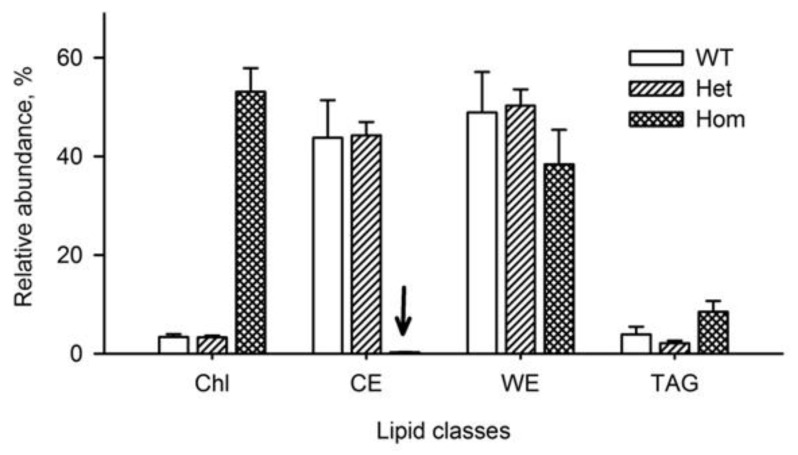
Effects of *Soat1* ablation on the balance of major Meibomian lipid classes. An almost complete suppression of the biosynthesis of cholesteryl esters was observed in *Soat1^−/−^* mice (black arrow). Normalized results are shown as mean ± standard deviation values. Sum of Chl, CE, WE, and TAG for each genotype equals 100%.

The apparent Chl, CE, WE, and TAG balances were estimated for WT, *Soat1^+/−^*, and *Soat1^−/−^* mice (Figure 7). These lipids form the bulk of meibum [9,11]. It became evident that inactivation of *Soat1* caused an almost complete arrest of the biosynthesis of CE, and a massive accumulation of nonesterified Chl that replaced CE. The other groups of ML were affected to a much lesser extent—the apparent abundance of WE was somewhat decreased, while the TAG fraction almost doubled compared with WT mice. Overall, it was concluded that free Chl completely displaced CE in *Soat1-*null mice and became the most prominent lipid in meibum.

### 2.3. Inactivation of Soat1 Increased the Melting Temperature of Meibum

**Figure 8:**
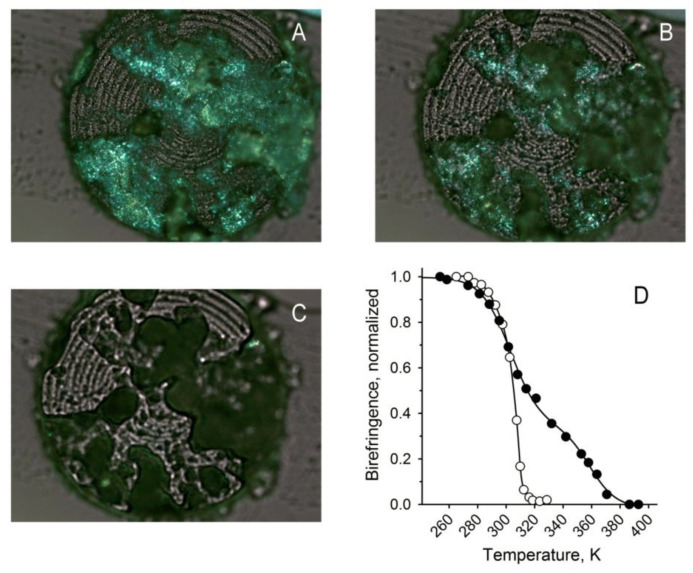
Effects of *Soat1* inactivation on melting characteristics of meibum. (**A**) Birefringent patterns of solidified *Soat1*-null mouse meibum at −25 °C; (**B**) partially melted *Soat1*-null meibum at +50 °C; (**C**) almost completely melted, amorphous, non-birefringent *Soat1*-null meibum at +125 °C; (**D**) melting curves of normal (open circles) and *Soat1*-null (closed circles) meibumDots—experimental values; solid lines—theoretical curves computed using Equation (2) (see Materials and Methods).

The changes in ML profiles caused by *Soat1* inactivation dramatically changed the physicochemical properties of meibum, specifically its thermotropic characteristics. Earlier, we used hot stage polarized light microscopy (HSPLM) to analyze normal and abnormal human meibum and mouse TP samples [22,23]. The same approaches have been employed in this study as well (Figure 8 and Supplementary Figure S2).

Inactivation of *Soat1* led to pathological changes in the mouse meibum. Meibum was expressed from the eyelids of three *Soat^−/−^* and three WT mice. First, a substantial increase in the melting temperature of the expressed mouse meibum was detected. Unlike WT meibum that started to melt at about 14 °C, reached 50% of melting at around 34 °C, and was completely liquefied by ~50 °C, the mutant meibum became 50% melted at about 50 °C and completely lost its birefringence only at above 100 °C (Figure 8A–C). Such a high melting temperature was characteristic of all tested *Soat1*-null samples.

Interestingly, melting of *Soat1*-null mouse meibum clearly had a multi-phase nature demonstrating at least two major transitions: A low-temperature transition between −20 and 50 °C (Transition I) and a high-temperature transition between 50 and ~120 °C. (Transition II), which implied the existence of low- and high-melting components in the samples. The experimental melting data were adequately represented by Equation (2) (Figure 8D). The low-temperature transition had a ***T*_1_** of 304 ± 4 K (or 31 °C; *n* = 3), while the high-temperature transition had ***T*_2_** of 359 ± 4 K (or 86 °C; *n* = 3), with their corresponding *Hill* cooperativity coefficients ***k*** and ***m*** being 39 and 29, correspondingly. These ***k*** and ***m*** coefficients were much lower than those for WT meibum—around 120 for both parameters–which underwent a solid-to-liquid transition in a much narrower temperature range and had a steeper melting curve than that of the *Soat1*-null meibum. Note the quality of fit of the data with an ***r^2^*** value of 0.999. The temperatures ***T*_1_** and ***T*_2_** for WT samples were, respectively, 289 ± 2 and 307 ± 1 K.

Another major difference between WT and *Soat1*-null specimens was the sample heterogeneity: Meibum specimens melted non-uniformly, with some areas melting at lower temperatures than the others. From a series of photographs taken at different temperatures (Figure 8 and Supplementary Figure S2), it became clear that intact meibum that was undergoing its first melting cycle had inclusions of high-melting birefringent material surrounded by lower-melting components. In addition, unlike WT meibum, the relative amounts of high-melting inclusions in *Soat1*-null meibum varied from sample to sample. This and, possibly, some other factors caused noticeable sporadic fluctuations in the melting behavior of *Soat1*-null samples and transition temperatures that approached ±3 °C for ***T*_1_** and ±4 °C—for ***T*_2_**. After the first melting cycle, domains with different transition temperatures partially, but not completely, merged. However, this process did not produce a uniformly melting lipid mixture: The second and subsequent melting cycles clearly demonstrated the presence of low- and high-melting components. 

In addition, all samples revealed amassing of a large number of non-melting, non-birefringent inclusion bodies similar to those described for human MGD specimens [22]. Those inclusions in human meibum were found to be of proteinaceous nature and were susceptible to Amido Black (a nonspecific protein stain), and anti-PanCK and anti-CK10 staining with corresponding antibodies. The nature of these inclusions in mouse meibum has not been evaluated in the current study and will be discussed in future publications.

## 3. Discussion

It has been proposed that MG maintain their lipid homeostasis via a network of synchronized biosynthetic reactions termed *meibogenesis* [7,11]. Meibum is formed of a large number of various lipids that include families of WE, CE, TAG, and other, more complex and unique, compounds [7,9]. We have reported that in healthy individuals and laboratory animals the inter-donor variability of ML profiles is very low [7,12,21] which is a prerequisite for ML to fulfill their physiological functions. After WE, which account for ~40% of ML, CE is the second largest group of Meibomian lipids that represents ~30% of the lipid pool (*w/w*). Meibomian CE are based on saturated and unsaturated C_10_ to C_34_ FA. Importantly, the makeup of CE in meibum demonstrated only subtle inter-donor variations in humans [12] and mice [21]. Recently, we reported that MG of humans highly expressed key genes of Chl biosynthesis, such as *ACAT1/ACAT2, DHCR7*, *DHCR24, FDFT1, HMGCR, HMGCS1/HMGCS2, LSS, MSMO1, SQLE,* and others, and so did MG of mice [7,11,12,23]. Genes that encode enzymes of CE biosynthesis via Chl esterification with FA—*SOAT1/Soat1, SOAT2/Soat2*, and *LCAT/Lcat*—were also identified. Of the latter three, *SOAT1/Soat1* emerged as the most highly expressed gene in MG of either species with the log(2) expression value of 17.2–17.5 in humans and about 18 in mice, while *LCAT* and *SOAT2* are expressed in human MG at much lower levels (around 7 and 5, correspondingly) [7,11,12]. These two factors—high levels of CE in meibum and high expression values in MG—made this gene a logical choice for our current mechanistic experiments.

Indeed, inactivation of *Soat1* resulted in a virtually complete loss of CE and Chl-OAHFA in meibum and accumulation of their precursor—free Chl—in large quantities, effectively replacing very, extremely, and ultra-long CE to become the major lipid component of abnormal meibum. Notably, short and medium chain CE with FA chains between C_10_ and C_16_ were still observed, but at very low levels, well below 3% of total Meibomian CE in WT mice. We hypothesize that the remaining CE were either products of *Soat2* or *Lcat*, or were derived from food. Thus, it is reasonable to conclude that the entire pool of Meibomian CE with FA of the C_21_–C_34_ family is produced entirely by SOAT1, with SOAT2/*Soat2* and LCAT/*Lcat* not playing any significant roles in the CE biosynthesis in MG. These observations complement earlier findings of Yagyu et al. [24] who observed a decline, but not complete obliteration of CE in ACAT1 (SOAT1)-deficient mice, leaving room for SOAT2- and LCAT-derived CE. However, our data clearly indicate that typical Meibomian CE with FA chains in the C_21_–C_34_ range are biosynthesized solely by SOAT1.

Accumulation of Chl in meibum of *Soat1*-null animals led to a noticeable change in the fluidity of the secretion: Meibum became stagnant in the central ducts, could not easily flow out of the orifices, induced their pouting, formed rod-like protrusions from the orifices, accumulated around the eyes in solidified form, and broke apart into smaller rice grain-shaped pieces while being expressed from the glands (Figure 1). This excessive rigidity of meibum, which is normally much more fluid [22,25], can only be attributed to disproportionately high accumulation of free Chl and decline in CE (Figure 3). Indeed, the melting temperature of pure Chl is about 148 °C (https://pubchem.ncbi.nlm.nih.gov/compound/cholesterol) meaning that it is highly rigid at physiological temperatures. Melting temperatures of individual CE, on the other hand, vary depending on the structures of their FA residues, but are usually much lower than that of Chl [26]. A drop in melting temperature of CE compared with Chl is caused by a looser, more disorganized packing of CE [27], especially unsaturated or branched, in solidified form. Thus, it seems plausible that enrichment of meibum with high-melting free Chl in lieu of lower-melting CE was the driving force behind the changes in the thermotropic characteristics of meibum and made it much more solid at physiological temperatures. 

However, the effect of Chl accumulation in meibum of *Soat1*-null mice has another aspect that needs to be discussed: Complex mixtures composed of various lipid species often do not form ideally mixed, uniform blends, but rather produce a range of domains that are highly enriched with one of the components, especially when that component is present in excess of its miscibility levels with other components of the mixture. This effect of (quasi)-two-dimensional phase separation of lipids in complex lipid mixtures is known from a number of in vitro studies (e.g., [28]). In case of *Soat1*-null meibum, Chl can be such a component. Indeed, free Chl that represents about 30% of ML in mutant mice may form Chl-enriched domains and be detectable in HSPLM experiments as a domain with unique characteristics, such as a much higher melting temperature. Characteristically, free Chl forms Chl-enriched domains in atherosclerotic plaques [29,30] which melt at much higher temperatures than CE found in the same loci [29]. In addition, Chl molecules aggregate to form rigid microcrystals instead of much more fluid and less dense micelles in lesions [30] and even in diluted aqueous solutions [31]. Thus, it is plausible that the high-melting components that are present in *Soat1*-null meibum (Figure 8 and Supplementary Figure S1) are indeed Chl-enriched domains. These, or similar, lipid aggregates might be one of the sources of the “sand in the eye” sensation that is often reported to physicians by dry eye/MGD patients, and could lead to ocular surface abrasions due to their rigidity, quantity, and size.

Lastly, CE-depleted meibum lost its ability to form thin, but continuous, layers in the conditions of HSPLM experiments and became highly fragmented after the first melting–cooling cycle (not shown). This can be interpreted as an indication of its diminished ability to form the tear film lipid layer and protect the eye. The mechanism of such a change is not known at this time and is to be evaluated in future experiments.

**Figure 9:**
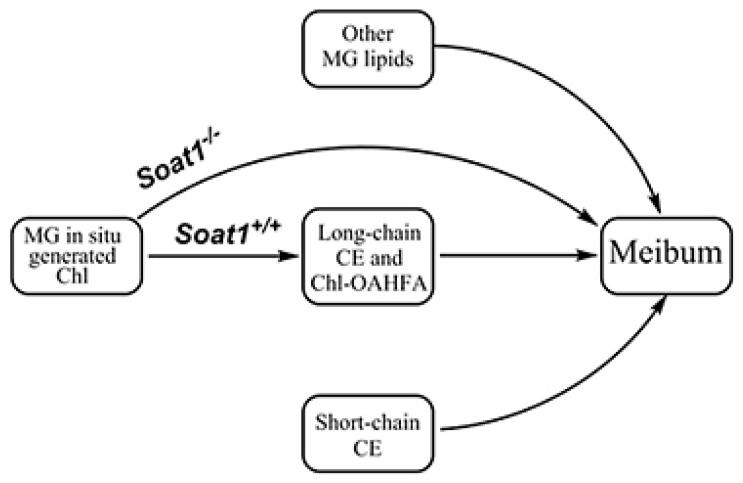
Role of *Soat1* in the biosynthesis of cholesteryl esters in Meibomian glands.

In conclusion, inactivation of *Soat1* confirmed its previously predicted critical role in meibogenesis [7,11,12,21,23] and led to an almost complete elimination of MG-derived CE and Chl-OAHFA (Figure 9). This change in meibum caused dramatic shifts in the ocular phenotype of *Soat1*-ablated mice that resembled many features characteristic of human MGD. Therefore, up- and/or downregulation of *Soat1*/SOAT1 may be influential factors that need to be considered when studying the etiology of these, and other, ocular surface diseases.

## 4. Materials and Methods

### 4.1. Reagents

Lipids standards were obtained from Sigma–Aldrich/MilliporeSigma (St. Louis, MO, USA) and Nu-Chek Prep., Inc. (Elysian, MN, USA). Organic solvents (acetonitrile, iso-propanol, chloroform, and methanol) for lipid extraction and analyses were of either HPLC or mass spectrometry grade. The solvents were obtained from Sigma–Aldrich, Thermo Fisher (Waltham, MA, USA), and/or Burdick and Jackson (Muskegon, MI, USA). Formic acid and ammonium formate were of >99.99% purity (from Sigma–Aldrich).

### 4.2. Animals and Animal Procedures

All animal procedures that were used in this study were approved by the Institutional Animal Care and Use Committee of the University of Texas Southwestern Medical Center (UTSW) and were conducted in accordance with the Association for Research in Vision and Ophthalmology (ARVO) Statement for the Use of Animals in Ophthalmic and Vision Research. *Soat1^+/−^* mice on a C57BL/6J background were purchased from the Jackson Laboratory (B6.129S4-*Soat1^tm1Far^*/Pgn; stock #007147; Bar Harbor, ME, USA). The targeted mutation resulted in *Soat1*-null mice which have no SOAT1 protein detected by immunoblotting samples from preputial gland, ovaries, and adrenals [32].

The animal colonies were maintained and bred in the Animal Research Center (ARC) of UTSW under constant supervision of ARC staff members (technicians and veterinarians) on a 12-h light/dark cycle with ad libitum access to food and water on the 2016 Teklad global 16% protein rodent diet (from Envigo, Indianapolis, IN, USA). 

Mice were genotyped using three primers described in the Jackson Laboratory protocol #24675 (all sequences 5′→3′): Primer 18,958 (TGC TGA CGT CTT CCT GTG TC, common), primer 18,959 (GAG CTG TTG GG AGT AGG TG, wild-type reverse), and primer oIMR6218 (CCT TCT ATC GCC TTC TTG ACG, mutant reverse), which all provided the expected bands 400 bp (for mutant) and 240 bp (for wild type). 

Evaluation of mouse ocular features was conducted using a slit lamp model BQ 900 (from Haag–Streit USA, Inc., Mason, OH, USA) as described recently [14]. TP samples were collected for analyses using a Zeiss Stemi 508 Stereo microscope (Carl Zeiss, Oberkochen, Germany) as described earlier [14]. Treated mice were euthanized using inhalant isoflurane followed by cervical dislocation. Briefly, four TP from each mouse were excised from the eyelids, dissected free from epidermis, and placed in HPLC-style glass sample vials filled with ~0.5 mL of chloroform:methanol = 2:1 solvent mixture (*v/v*; CM21) for overnight extraction in a fridge. Then, the lipids were repetitively extracted with 3 × 1 mL of the same solvent mixture, the extracts were combined, the solvent was evaporated at 37 °C under a gentle stream of nitrogen, and the remaining lipid material was re-dissolved in iso-propanol (between 0.2 and 0.5 mL per 4 TP). The samples were sealed and stored at −20 °C until analyses. Samples of meibum were obtained by expressing the secretion from the orifices, collecting the samples with a microspatula, and dissolving the specimens in the CM21 solvent mixture. For RP UPLC/APCI MS analyses, the CM21 mixture was evaporated and the samples were re-dissolved in iso-propanol as described above. Five *Soat1^−/−^*, seven *Soat1^+/−^*, and seven WT mice were tested. Statistical aspects of the analyses (necessary numbers of biological samples and technical replicas, reproducibility of the analyses, methods of sample quantitation, etc.) are described in detail in our recent publications [7,12,14,21] and are not repeated here to avoid duplication in reporting.

### 4.3. Analytical Instrumentation and Procedures

Meibomian lipids and lipid standards were analyzed in MS^E^ mode using a high-resolution quadrupole Time-of-Flight Synapt G2-Si mass spectrometer equipped with an IonSabre-II APCI ion source and a LockSpray unit (all from Waters Corp., Milford, MA, USA). Chromatographic separation of lipids was performed on an Acquity M-Class binary ultra-high performance liquid chromatograph (also from Waters), in RP mode, using an acetonitrile/iso-propanol gradient as described before [21] at a 20 µL/min flow rate at 30 °C. A Waters Acquity UPLC C18 BEH column (1 × 100 mm, 1.7 µm) was used.

The chromatographic data were analyzed as total ion chromatograms and EIC: The first approach was used to assess the overall quality of the samples, while the later provided information about specific analytes.

Chl and CE profiles in the samples were assessed as follows. Both types of lipids are known to produce a common analytical ion of dehydrated and protonated cholesterol with a theoretical *m/z* value of 369.3521 (a neutral loss of water for Chl and a neutral loss of FA for CE). In chosen experimental conditions, monitoring this ion provided a convenient way of detecting not only Chl itself, but also other Chl-containing compounds such as CE and Chl-OAHFA [12,21,23]. Additionally, typical adducts (such as proton, ammonia, sodium, and potassium ones) of intact lipids were also analyzed, when needed. The choice of the MS^E^ mode of detection allowed us to assess low and high energy fragmentation patterns of analytes, which facilitated their identification.

Unlike CE, WE do not have a particular common analytical ion, unless the focus is on their FA moieties, in which case one can monitor all WE that have a particular FA in their structure. Thus, we resorted to a tested and proven approach based on monitoring their (M + H)^+^ adducts [10,21,23].

Finally, TAG were detected as (M + H)^+^ and (M − FA + H)^+^ ions, as described in our recent publications [21,33].

Melting characteristics of expressed meibum were evaluated using HSPLM as described earlier ([22,23]. A Nikon Eclipse 50i POL microscope (Nikon Instruments, Melville, NY, USA) with a Nikon DS-Fi1 color digital camera, and an LTS420 heating-cooling stage (from Linkam Scientific Instruments, Waterfield, UK) were used. Birefringence of meibum in liquid crystal state (***I_br_***) was monitored and its intensity was recorded at different temperatures (***T***). Data plotted as ***I_br_*** vs. ***T*** were analyzed in NIS-Elements BR software package (also from Nikon). As meibum melts in a non-linear, cooperative fashion [22,23] with at least three clearly defined aggregation states, the following transformations were considered:*Crystal (solid, birefringent)* →*Liquid crystal (partially melted, birefringent*;*possibly, 2 forms—smectic and cholesteric)* →*Isotropic (fluid, non-birefringent)*

The melting curves were analyzed using (a) numeric differentiation of their spline approximations (an unbiased approach that is not based on any specific mechanism), and (b) a two-transition temperature, Hill-type Equation (2):

***I_br_*** = ***A*** − (***B*** × ***T^k^***)/(***T*_1_*^k^*** + ***T^k^***) − (***C*** × ***T^m^***)/(***T*_2_*^m^*** + ***T^m^***) (2)

where ***I_br_*** is birefringence at the current temperature ***T*** (in Kelvins), ***A*** is birefringence of fully crystallized meibum (equals 1, if normalized), ***B*** and ***C*** are respective contributions of two birefringent, liquid-crystal forms of meibum, or its two major “domains” (more than 0, but less than 1, when normalized), ***T*_1_** and ***T*_2_** are two phase transition temperatures for forms ***B*** and ***C***, while ***k*** and ***m*** are the Hill cooperativity coefficients (unitless). The parameters of Equation (1) were determined using a nonlinear curve fitting routine. Note that it is not currently known if forms ***B*** and ***C*** are two different aggregation forms of the same lipid mixture (such as smectic and cholesteric), or two “domains” with different lipid compositions that coexist in meibum. However, for the purpose of this paper they have been considered as two different “domains” in the broadest sense of the word. 

### 4.4. Data Computation and Statistical Analyses

Experimental results were analyzed using a MassLynx software package v.4.1. (from Waters) and SigmaStat v.3.5 (from Systat Software, Inc., San Jose, CA, USA). The elemental CHO formulas of the analytes were computed using the EleComp routine of the MassLynx software package. Routinely, the accuracy of the analyses was in the 3 to 10 mDa range, which, in combination with MS^E^, allowed for reliable identification of unknown analytes.

## Data Availability

All the data that are pertinent to the discussion are included in the paper.

## Supplementary Materials

The following are available online at https://www.mdpi.com/1422-0067/22/4/1583/s1, Figure S1: An extracted ion chromatogram of free cholesterol (Chl) and cholesteryl esters (CE) of a representative wild type mouse tarsal plate extract, Figure S2: Melting of a Soat1-null meibum sample as recorded during a HSPLM experiment.
