# Supplementary material for: Depletion of Cholesteryl Esters Causes Meibomian Gland Dysfunction-Like Symptoms in a Soat1-Null Mouse Model
Source: Int J Mol Sci. 2021 Feb 4;22(4):1583. doi: 10.3390/ijms22041583 (PMC7915537; doi:10.3390/ijms22041583)
Supplement: Supplementary file 1 [file ijms-22-01583-s001.pdf]

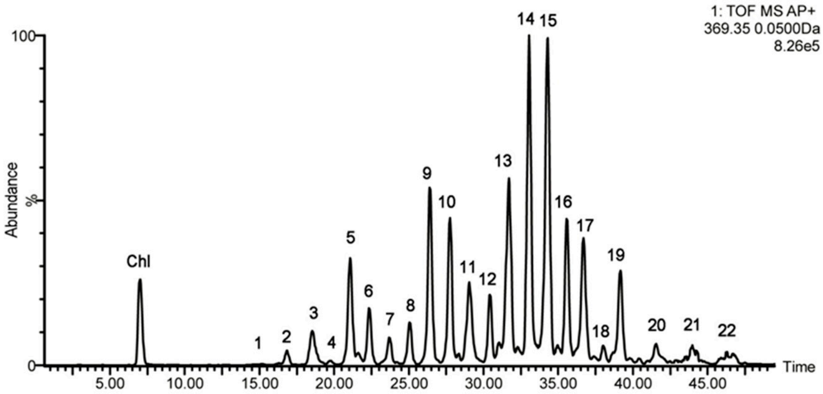

Figure S1. An extracted ion chromatogram of free cholesterol (Chl) and cholesteryl esters (CE) of a representative wild type mouse tarsal plate extract. A common analytical ion *m/z* 369.35 was used to detect the analytes. The following assignments for CE were made based on the *m/z* values and fragmentation patterns of their $(M + H)^+$, $(M + K)^+$ and $(M + Na)^+$ adducts: $C_{10:0}$ (1); $C_{12:0}$- and $C_{14:1}$-CE (2); $C_{14:0}$ and $C_{16:1}$ (3); $C_{15:0}$ (4); $C_{16:0}$- and $C_{18:1}$-CE (5); $C_{17:0}$-CE (6); $C_{18:0}$- and $C_{20:1}$-CE (7); $C_{19:0}$-CE (8); $C_{20:0}$- and $C_{22:1}$-CE (9); $C_{21:0}$-CE (10); $C_{22:0}$- and $C_{24:1}$-CE (11); $C_{23:0}$-CE (12); $C_{24:0}$- and $C_{26:1}$-CE (13); $C_{25:0}$-CE (14); $C_{26:0}$- and $C_{28:1}$-CE (15); $C_{27:0}$-CE (16); $C_{28:0}$- and $C_{30:1}$-CE (17); $C_{29:0}$ (18); $C_{30:0}$- and $C_{32:1}$-CE (19); $C_{34:1}$-CE (20); cholesteryl esters of (*O*)-acyl-ω-hydroxy fatty acids (20-22).

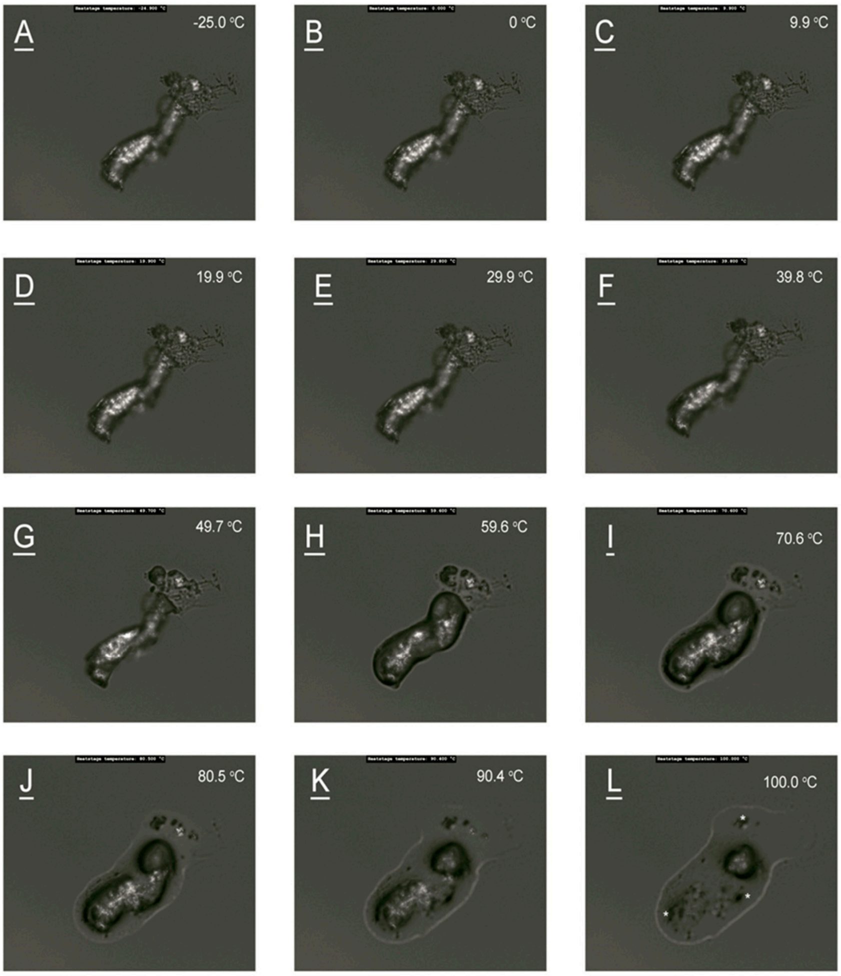

Figure S2. Melting of a *Soat1*-null meibum sample as recorded during a HSPLM experiment. Sample temperatures rose from −25 °C to +100 °C with a rate of 2 °C/min. Bright structures visible within the meibum sample are birefringent lipid aggregates. Note that some aggregates did not melt even at the highest temperature. Dark, non-melting, non-birefringent aggregates of presumably proteinaceous nature are labeled with white asterisks in Panel L.
